# Culture of human pluripotent stem cells using completely defined conditions on a recombinant E-cadherin substratum

**DOI:** 10.1186/1471-213X-10-60

**Published:** 2010-06-02

**Authors:** Masato Nagaoka, Karim Si-Tayeb, Toshihiro Akaike, Stephen A Duncan

**Affiliations:** 1 Department of Cell Biology, Neurobiology and Anatomy, Medical College of Wisconsin, 8701 Watertown Plank Road, Milwaukee, WI 53226, USA; 2 Department of Biomolecular Engineering, Graduate School of Bioscience and Biotechnology, Tokyo Institute of Technology, 4259 Nagatsuta-cho, Midori-ku, Yokohama 226-8501, Japan

## Abstract

**Background:**

To maintain pluripotency of human embryonic stem (huES) cells in feeder-free culture it has been necessary to provide a Matrigel substratum, which is a complex of poorly defined extracellular matrices and growth factors derived from mouse Engelbreth-Holm-Swarm sarcoma cells. Culture of stem cells under ill-defined conditions can inhibit the effectiveness of maintaining cells in a pluripotent state and reduce reproducibility of differentiation protocols. Moreover recent batches of Matrigel have been found to be contaminated with the single stranded RNA virus, Lactate Dehydrogenase Elevating Virus (LDEV), raising concerns regarding the safety of using stem cells that have been cultured on Matrigel in a therapeutic setting. To circumvent such concerns, we attempted to identify a recombinant matrix that could be used as an alternative to Matrigel for the culture of human pluripotent stem cells. huES and human induced pluripotent stem (hiPS) cells were grown on plates coated with a fusion protein consisting of E-cadherin and the IgG Fc domain using mTeSR1 medium.

**Results:**

Cells grown under these conditions maintained similar morphology and growth rate to those grown on Matrigel and retained all pluripotent stem cell features, including an ability to differentiate into multiple cell lineages in teratoma assays. We, therefore, present a culture system that maintains the pluripotency of huES and hiPS cells under completely defined conditions.

**Conclusions:**

We propose that this system should facilitate growth of stem cells using good manufacturing practices (GMP), which will be necessary for the clinical use of pluripotent stem cells and their derivatives.

## Background

The generation of human embryonic stem (huES) cells and induced pluripotent stem (hiPS) cells has been well-established [1-3]. Pluripotent cells have the potential to be used for cell therapy as well as the study of human disease and development and so offer a resource that could have substantial biomedical impact [4]. Initially, the sustained culture of pluripotent stem cells required growth on a layer of mouse embryonic fibroblasts (MEF), which presumably provide factors that sustain cell pluripotency and viability [1]. Recently, a variety of culture media have been established that can circumvent the need for feeder cells as long as the cells are cultured on a substratum, which is usually Matrigel [5]. Xu *et al *examined various purified extracellular matrix substrates for their ability to support culture of huES cells and found that Matrigel can maintain the pluripotency of huES cells efficiently [6]. Therefore, Matrigel has been used for feeder free culture of huES cells in most studies to date.

Matrigel is a purified gel matrix from Engelbreth-Holm-Swarm sarcoma cells that consists of a mixture of extracellular matrices, proteoglycans, and growth factors [7-9]. It is highly biologically active and closely resembles basement membrane in both consistency and activity [10]. In addition to facilitating the culture of pluripotent stem cells, Matrigel is also used to induce cell differentiation, facilitate invasion of cancer cells, increase tumor growth, and has been used extensively to support duct formation and angiogenesis. Although acting as a viable substitute for basement membrane, Matrigel is a relatively impure preparation, with significant lot to lot variability. For the culture of huES cells, growth factor-reduced Matrigel is used [11]. Although an improvement over the standard preparations, the levels of growth factors that remain are substantial and are likely to impact the reproducibility of the culture and controlled differentiation of pluripotent stem cells in general. Additional concerns have been raised recently due to the widespread distribution of preparations of Matrigel that were contaminated with Lactate Dehydrogenase Elevating Virus (LDEV) [12]. Such contaminations raise serious safety issues if pluripotent stem cells are to be used for autologous cell therapy.

There has been considerable discussion over the development of ideal culture conditions for huES cells using defined matrix, defined media supplemented with recombinant proteins, together with the elimination of xenogeneic components, such as FBS or feeder cells. However, significant difficulties remain in developing fully defined huES cell culture conditions, including the need for a suitable cell surface matrix. In an attempt to overcome problems associated with the use of Matrigel, we sought to examine the feasibility of using recombinant protein as a defined substratum that could support the culture of pluripotent human stem cells. E-cadherin, a Ca^2+^-dependent cell-cell adhesion molecule [13,14], is essential for intercellular adhesion and colony formation of mouse embryonic stem cells [15,16]. Several reports suggest that E-cadherin levels in huES cells decrease during differentiation [17,18] and so high expression of E-cadherin is characteristic of undifferentiated pluripotent stem cells. We have previously reported that mouse embryonic stem cells can be successfully maintained on surfaces coated with a fusion protein consisting of the E-cadherin extracellular domain and the IgG Fc domain (E-cad-Fc) [19]. On E-cad-Fc-coated plates mouse ES cells do not form colonies but do retain pluripotency and can be used to generate germline-competent chimeric mice [19,20]. We, therefore, examined whether human E-cad-Fc could substitute for Matrigel and support pluripotency of huES and hiPS cells using completely-defined culture conditions.

## Results

### Maintenance of huES cells on an hE-cad-Fc-coated surface under standard culture conditions

**Figure 1:**
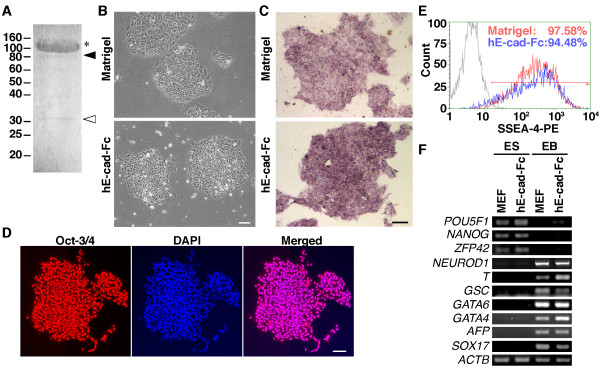
**Human ES cells maintain pluripotency on hE-cad-Fc-coated plates**. (A) Purified hE-cad-Fc was separated by SDS-PAGE, and three bands were observed by CBB staining. TOF-MASS analysis revealed that the major 120 kDa band (*) represents full-length hE-cad-Fc, while the 90 kDa band (black arrowhead) was E-cadherin alone and the 30 kDa band (white arrowhead) was the IgG Fc protein alone. (B) Phase contrast micrographs showing the morphology of H9 huES cells cultured on polystyrene surfaces coated with Matrigel or hE-cad-Fc in MEF conditioned medium 7 days after seeding. (C) Histochemistry revealing alkaline phosphatase activity (purple) in H9 cells cultured on Matrigel or on hE-cad-Fc after 68 passages. (D) Immunocytochemistry to detect OCT3/4 (red) in H9 cells cultured on hE-cad-Fc-coated dishes for 13 passages (90 days). Nuclei were counterstained with DAPI. Scale bar indicates 100 μm. (E) The expression of SSEA-4 was analyzed by FACS after 69 passages on hE-cad-Fc. (F) H9 cells were cultured on feeder cells (MEF) or on hE-cad-Fc-coated plates for 13 passages (90 days) (ES), and then in suspension to form embryoid bodies for 21 days (EB). The expression of mRNAs characteristic of the undifferentiated state and specific cell lineages was then identified by RT-PCR.

We initiated our studies by examining the purity of hE-cad-Fc produced as described in the methods section. Purified hE-cad-Fc was subjected to SDS-PAGE followed by CBB staining, which detects nanogram levels of proteins. These analyses revealed that the purified fraction of hE-cad-Fc consisted of three protein species that migrated as 120 kDa, 90 kDa and 30 kDa bands (Figure 1A). Proteins present in each band were then identified by TOF-MASS analysis. The predominant 120 kDa band represented the expected hE-cad-Fc fusion protein, while the 90 kDa and 30 kDa species contained only hE-cadherin and IgG Fc as separate components, respectively. These data demonstrate that we are working with a highly purified fraction of recombinant hE-cad-Fc protein that is essentially free of any relevant biological contaminants.

First, we asked whether hE-cad-Fc-coated culture dishes could support propagation and maintain pluripotency of huES cells under conditions that support ES cell culture on Matrigel. In our laboratory H9 (WA09) ES cells are routinely passaged on a feeder layer of mitotically inactive embryonic fibroblasts (MEFs). Cells were harvested by manual dissection initially followed by trypsin digestion to produce a single cell suspension, transferred to plates coated with either Matrigel or hE-cad-Fc and grown in MEF-conditioned medium (MEF-CM). Since trypsin treatment reduced cell viability on both matrices, we used Accutase from the 2nd passage onward. After 7-days in culture the huES cells grown on Matrigel or hE-cad-Fc were found to be morphologically indistinguishable (Figure 1B) and this morphology was maintained for >13 passages using Accutase under both conditions. In both cases the cells had formed densely packed colonies and the individual cells had a high nuclear to cytoplasmic ratio that is typical of huES cells. We next determined whether expression of pluripotency markers could be maintained following extended culture of huES cells on hE-cad-Fc. Cells were cultured for ≥90 days on either Matrigel or hE-cad-Fc, with the cells being passaged on a weekly basis. Figures 1C and 1D show that H9 huES cells cultured on hE-cad-Fc-coated dishes maintained expression of alkaline phosphatase activity (68 passages: Figure 1C) and expression of Oct-3/4 (13 passages: Figure 1D) which was comparable to cells cultured on Matrigel. As shown in Figure 1E, FACS analyses determined that >94% of cells expressed SSEA-4, irrespective of whether Matrigel or hE-cad-Fc was used as a substrate. RT-PCR analyses revealed that huES cells cultured either on MEFs or on hE-cad-Fc maintained expression of *POU5F1 *(*OCT-3/4*), *NANOG *and *ZFP42 *(*REX-1*) mRNAs, which are characteristic of pluripotent stem cells (Figure 1F). Like control H9 cells cultured on Matrigel, cells cultured using hE-cad-Fc were also able to differentiate into multiple cell lineages after formation of embryoid bodies. Figure 1F shows that mRNAs that are characteristic of ectoderm (*NEUROD1*), mesoderm (*T/BRACHYURY *and *GSC*) and endoderm (*GATA4*, *GATA6*, *AFP *and *SOX17*) could be identified in embryoid bodies from H9 ES cells that had been cultured on either MEFs or hE-cad-Fc-coated surfaces. Importantly, none of these mRNAs were detected in huES cells cultured on hE-cad-Fc plates before embryoid body formation. From these data we conclude that hE-cad-Fc can substitute for Matrigel in supporting the extended culture and pluripotency of huES cells.

### huES cells cultured under completely defined conditions

**Figure 2:**
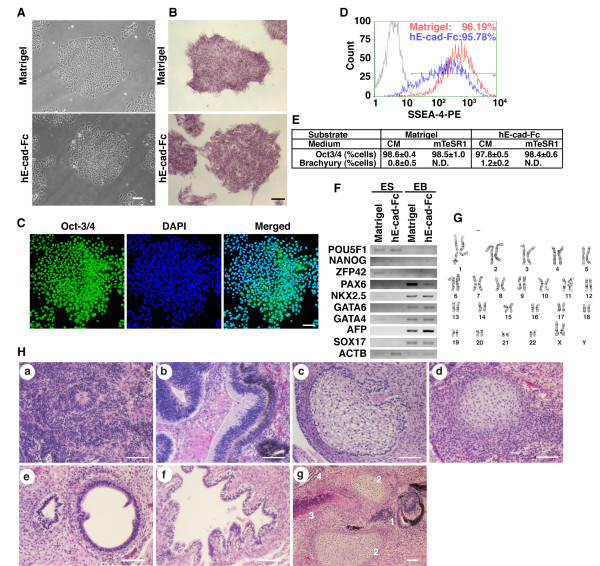
**Maintenance of human ES cells under the completely chemically defined condition**. (A) Phase contrast micrographs showing H9 cells cultured with mTeSR1 media for 35 passages on hE-cad-Fc or 11 passages on Matrigel. Scale bars indicate 100 μm. (B) Alkaline phosphatase activity in H9 cells cultured on Matrigel or on hE-cad-Fc under defined conditions (53 passages). (C) H9 cells cultured on hE-cad-Fc-coated surface in mTeSR1 media for 36 passages were identified by immunocytochemistry using an anti-Oct-3/4 antibody. Nuclei were counterstained with DAPI. Scale bar indicates 100 μm. (D) The expression of SSEA-4 was analyzed by FACS after 53 passages on hE-cad-Fc. (E) Immunocytochemical analysis of protein expression in human ES cells cultured in various conditions. The data represent means ± SD of three individual experiments. (F) The expression of genes characteristic of the undifferentiated state was analyzed by RT-PCR, as in Figure 1F. (G) Karyotyping of H9 cells cultured for 30 passages on a hE-cad-Fc-coated surface with mTeSR1 media. (H) Characterization of teratomas from H9 cells cultured on an hE-cad-Fc-coated surface. Hematoxylin and eosin staining of paraffin sections through teratomas identified the differentiation huES cells into various tissues, including immature neuroblastic tissue with neuronal rosettes (a), neuroepithelium with pigment (b), immature sebaceous tissue (c), cartilage (d), columnar epithelium (e), and gut-like epithelial structures (f). Panel g contains neural tissue (1), cartilage (2), bone parenchyma (3), and epithelial tissue (4). Bar indicates 100 μm.

In the previous experiments the culture media that supported huES cell growth on the hE-cad-Fc surface contained 20% knockout serum replacement that was conditioned by MEFs. Recent studies have described a culture medium, mTeSR1, which consists solely of defined components that can support the derivation and culture of huES cells on Matrigel [21]. We, therefore, examined whether mTeSR1 could support the culture of huES cells on an hE-cad-Fc-coated surface (Figure 2). Cells were transferred to either Matrigel or hE-cad-Fc-coated plates and cultured using mTeSR1 (Figure 2A) and then examined for expression of alkaline phosphatase activity (53 passages) and Oct-3/4 (35 passages) (Figures 2B and 2C). The maintenance of an undifferentiated phenotype was confirmed by flow cytometry, which revealed that 95% of cells grown on hE-cad-Fc continued to express SSEA-4 (Figure 2D) and 98% of cells expressed OCT3/4, which was statistically indistinguishable from cells cultured on Matrigel or on hE-cad-Fc plates with MEF-conditioned medium. Maintenance of expression of pluripotency genes was also confirmed by RT-PCR analyses (Figure 2F), which identified expression of *POU5F1 *(*OCT3/4*), *NANOG*, and *ZFP42 *mRNAs. Similar results were also obtained when H1 huES cells were cultured on hE-cad-Fc-coated plates under defined conditions (data not shown).

Extensive culture under feeder-free conditions, including on Matrigel, commonly results in accumulation of chromosomal abnormalities [22]. Rosler *et al *reported that up to 20% of H9 cultures grown using feeder-free conditions on a Matrigel-coated surface demonstrated genetic abnormalities and that trisomy 20 is the most frequent chromosomal defect found in H1, H7 and H9 cells [22]. We therefore examined the karyotype and performed g-banding analyses of H9 cells that had been cultured on the hE-cad-Fc-coated surface after 37 consecutive passages (>60 passages in total). No chromosomal rearrangements were identified. Of the 20 cells examined 19 had a normal diploid karyotype (Figure 2G); however, one aneuploid cell was found to have trisomy 20. Fluorescent In Situ Hybridization analysis (FISH) was therefore performed on 200 cells using probes to detect chromosomes 7 and 20. Analyses of the 200 cells revealed that all cells had a normal copy number, suggesting that the identification of the trisomy 20 by cytogenetic analysis may have been an experimental artefact or that it is an extremely rare occurrence. These data imply that even after extensive culture, the hE-cad-Fc substrate can support a stable population of pluripotent stem cells. Nevertheless, the possibility of low numbers of cells putatively harbouring trisomy 20 emphasises the need to routinely examine the karyotype of all pluripotent stem cell cultures, regardless of growth conditions.

We also used RT-PCR analyses to assess the extent of cell differentiation following extended culture in mTeSR1 on hE-cad-Fc using both immunocytochemistry and RT-PCR analysis (Figure 2E and 2F). Although Brachyury, an early marker of mesendodermal cell differentiation, was identified by immunostaining in approximately 1% of cells cultured in MEF conditioned medium on either surface (Figure 2E), this differentiation marker was not found to be expressed in cells maintained in mTeSR1 regardless of whether the surface consisted of Matrigel or hE-cad-Fc. Similarly, low levels of mRNAs that are characteristically found in mesoderm, ectoderm, and endoderm, could be detected by RT-PCR in cells cultured on Matrigel, but not when cells were cultured on hE-cad-Fc plates (Figure 2F). When cells cultured in mTeSR1 medium were removed from either the Matrigel or hE-cad-Fc plates and grown in suspension, they retained the capacity to form embryoid bodies and express lineage-enriched genes (Figure 2F), indicating that huES cells could differentiate into multiple cell types when cultured under these highly defined conditions. Finally, we examined whether pluripotency of huES cells was retained after an extended 13 passages in mTeSR1 on hE-cad-Fc coated plates by following their ability to generate teratomas in Rag2^-/-^Il2rg^-/- ^mice. Nine weeks after transplantation, teratomas had formed and histological analyses revealed the presence of cells from all three germ layers (Figure 2H; panels a-g).

**Figure 3:**
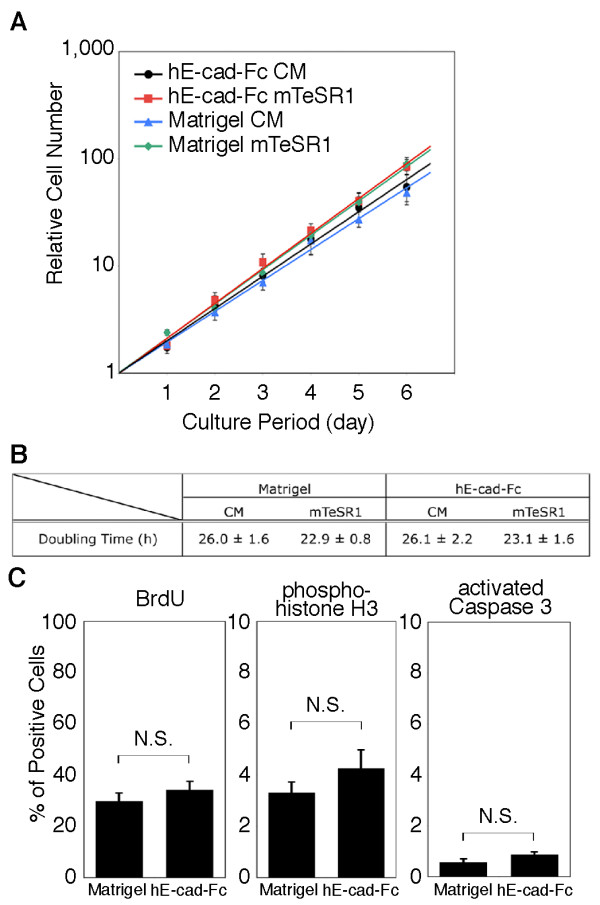
**Proliferation of human ES cells on an hE-cad-Fc-coated surface**. (A) The proliferative activity of ES cells was evaluated in surfaces coated with either Matrigel or hE-cad-Fc-coated. H9 cells were seeded on Matrigel- or hE-cad-Fc-coated dishes and cultured in MEF conditioned media (CM) or mTeSR1, and the cell number was counted after staining with WST-1 reagent. The data indicate means ± SEM of individual experiments (n = 5). (B) Growth characteristics of human ES cells cultured in various conditions. The data represent means ± SEM of five individual experiments. (C) The number of BrdU-, phospho-histone H3-, and activated caspase 3-positive cells were determined by immunohistochemistry after 2 days culture on a Matrigel- or hE-cad-Fc-coated surface. The data represent means ± SEM of five individual experiments. N.S. = not statistically significant.

Finally, we compared the proliferative capacity of huES cells cultured either on hE-cad-Fc- or Matrigel-coated surfaces in either MEF conditioned medium or mTeSR1. Cell counting (Figure 3A), measurement of doubling time (Figure 3B), and counting numbers of cells in S-phase (BrdU-labeling) and mitosis (phospho-histone H3 labeling) (Figure 3C), revealed that both Matrigel and hE-cad-Fc supported cell proliferation at similar rates; although, cells cultured in mTeSR1 grew slightly faster (doubling time = ~23 hours) than those grown in MEF conditioned medium (doubling time = ~26 hours) regardless of the substratum. Immunocytochemistry to detect the presence of activated caspase 3 revealed that the numbers of cells undergoing cell death were also statistically indistinguishable between huES cells grown on Matrigel and those on hE-cad-Fc (Figure 3C). Cumulatively, these data support the conclusion that hE-cad-Fc provides a substratum that facilitates the culture of huES cells under highly defined culture conditions.

### Plating efficiency of huES cells on an hE-cad-Fc-coated surface

**Figure 4:**
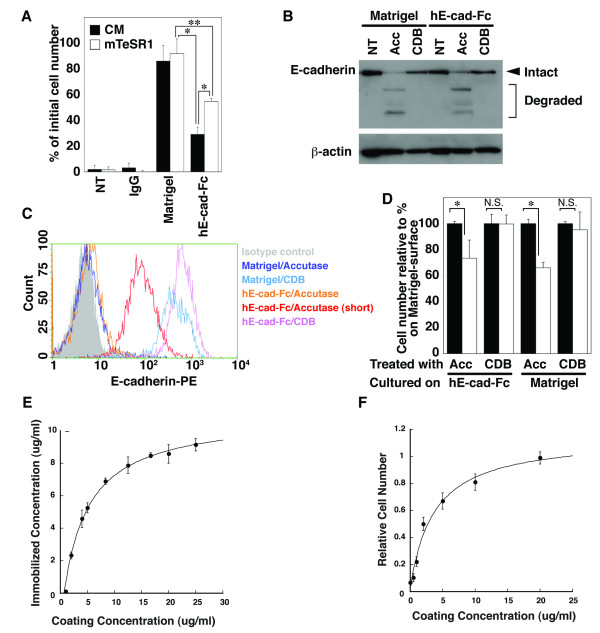
**Adhesion of H9 cells on an hE-cad-Fc coated surface**. (A) Number of adherent H9 cells was determined for hE-cad-Fc-coated and Matrigel-coated dishes following incubation for 3-hours in conditioned media (black) or mTeSR1 (white). The data are presented as means ± SD of 3 independent experiments. *: *p *< 0.01, **: *p *< 0.05. (B, C) E-cadherin was identified by immunoblot (B) or by FACS (C) after collecting cells by scraping (NT), Accutase (Acc) or enzyme free Cell Dissociation Buffer (CDB). When cells were incubated for <5 mins in Accutase (short) (C) a subset of cells retained cell surface E-cadherin, which facilitated plating (A). (D) Adhesion of H9 cells onto the hE-cad-Fc-coated surface was restored by CDB treatment. H9 cells cultured on hE-cad-Fc- or Matrigel-coated surface were treated with Accutase (Acc) or CDB. The adhesion of cells onto hE-cad-Fc (open bar) or Matrigel (closed bar) was analyzed by WST-1 assay. The data are means ± SD of 3 independent experiments. *: *p *< 0.01, N.S.: non significant. (E) The concentration of immobilized hE-cad-Fc was analyzed by HRP-labelled hE-cad-Fc, and fitted to a Langmuir isotherm curve. The data are means ± SD of 3 independent experiments. (F) H9 cells were treated with CDB and seeded onto the indicated concentration of hE-cad-Fc-coated surface in mTeSR1 media. The number of adhered cells was counted by WST-1 reagent and compared with the cell number on 20 μg/ml of hE-cad-Fc. The data are means ± SEM of 6 independent experiments.

Matrigel is a complex mixture of extracellular proteins and so it seems likely that adherence of huES cells to this surface will involve interactions with multiple cell surface molecules. As a single protein component, the use of hE-cad-Fc as a substratum could potentially change the dynamics of the binding of huES cells to the plate. We, therefore, examined the efficiency through which huES cells could adhere to an hE-cad-Fc coated culture plate. huES cells passaged on a Matrigel-coated surface in MEF-conditioned medium were isolated using Accutase, resuspended in either MEF conditioned media or mTeSR1, then transferred to either uncoated dishes or dishes coated with IgG, Matrigel, or hE-cad-Fc. The number of cells that had adhered to the culture dish after 3 hrs was then measured by cell counting. As expected cells did not adhere to either uncoated dishes or to IgG-coated dishes. In contrast to Matrigel, where >80% of plated cells were found to adhere in either MEF-conditioned media or mTeSR1, the hE-cad-Fc-coated surface supported adherence of only 30% of cells using MEF-conditioned media and 50% using mTeSR1 (Figure 4A).

The observation that huES cells could adhere to an hE-cad-Fc-coated surface, albeit with reduced efficiency compared to Matrigel, but could not adhere to an IgG-coated surface, suggested that huES cells were likely to bind to E-cad-Fc-coated plates through interactions between cell surface E-cadherin and the E-cadherin domain of hE-cad-Fc. Since E-cadherin is often targeted by proteases, we postulated that the relative reduction in adherence of huES cells to an hE-cad-Fc-coated surface compared to Matrigel may reflect the degradation of cell surface E-cadherin by Accutase. The active ingredients of Accutase are proprietary; however, it has been marketed as having both protease and collagenolytic activities. To test whether Accutase resulted in the degradation of E-cadherin, cells were harvested from Matrigel and hE-cad-Fc-coated plates using either Accutase, enzyme-free Cell Dissociation Buffer, or by scraping, and the state of E-cadherin protein examined by immunoblotting (Figure 4B). When immunoblots were probed with an antibody that recognizes the carboxyl end of E-cadherin it revealed a single 120 kDa band in cells isolated by scraping or under enzyme free conditions. In contrast to these control cells, in cells collected using Accutase the same antibody recognized additional faster migrating bands, which presumably are products of Accutase-mediated proteolysis, in addition to small amounts of the full length E-cadherin. The loss of E-cadherin on the surface of huES cells following Accutase treatment was confirmed by FACS analysis using a phycoerythrin-conjugated anti-E-cadherin antibody (Figure 4C).

The observation that enzyme-free Cell Dissociation Buffer preserved full-length E-cadherin suggested that the efficiency of adhesion of huES cells to an hE-cad-Fc-coated surface could be improved if cells were collected using enzyme-free Cell Dissociation Buffer instead of Accutase. To test this, huES cells were cultured on either Matrigel or hE-cad-Fc-coated plates and harvested using Accutase or enzyme-free Cell Dissociation Buffer. Harvested cells were then plated onto new plates containing either Matrigel or hE-cad-Fc-coated surfaces and the number of adhesive cells were counted. Figure 4D shows that, as before, harvesting cells using Accutase reduced the number of cells adhering to the E-cadherin substratum compared to Matrigel. However, when huES cells were collected using enzyme-free Cell Dissociation Buffer there was no difference in the efficiency of plating cells on either Matrigel or an hE-cad-Fc-coated surface.

We finally determined the optimum concentration required for efficient cell plating. As shown in Figure 4E, the culture surface was saturated after application of hE-cad-Fc at 15 μg/ml. The number of H9 cells adhering to the hE-cad-Fc-coated surface was found to increase in proportion to the concentration of hE-cad-Fc used for coating, with 10-20 μg/ml hE-cad-Fc producing maximal H9 adherence (Figure 4F). These results indicate that the reduced adhesion of huES cells to an hE-cad-Fc-coated surface after Accutase treatment is likely due to the degradation of cell surface E-cadherin and that enzyme-free dissociation buffer can facilitate efficient passaging of huES cells grown on plates coated with 20 μg/ml hE-cad-Fc.

### Maintenance of human iPS cells on an hE-cad-Fc-coated surface without feeder cells

**Figure 5:**
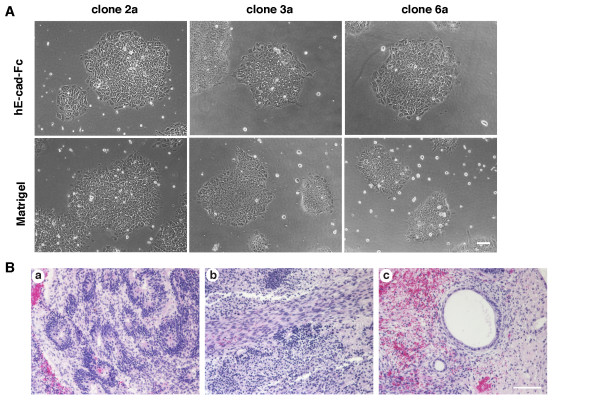
**Maintenance of human induced pluripotent stem cells on an hE-cad-Fc surface**. (A) Morphology of human iPS cell clones (2a, 3a and 6a) on surfaces coated with Matrigel or hE-cad-Fc. Scale bar indicates 100 μm. (B) Characterization of teratomas from iPS cells (clone 2a) cultured on an hE-cad-Fc-coated surface. Hematoxylin and eosin staining of teratomas showed the differentiation into various tissues, including immature neuroblastic tissue with neuronal rosettes (a), striated muscle (b), and columnar epithelium (c). Bar indicates 100 μm.

The determination that an hE-cad-Fc-coated surface could support pluripotency of huES cells suggested that the same would hold true for induced pluripotent stem cells generated from human fibroblasts. Three independent human iPS cells (hiPS 2a, 3a and 6a) were generated previously from foreskin fibroblasts and shown to be pluripotent [23]. The hiPS cells were maintained on a feeder layer of MEFs using the same methods as for huES cells. Three independent hiPS cell lines (2a, 3a and 6a) were cultured on a MEF feeder layer, manually scraped and then seeded onto dishes coated with hE-cad-Fc or Matrigel. All of the hiPS lines adhered to the hE-cad-Fc-coated surface, forming colonies indistinguishable from human ES cells (Figure 5A). One hiPS cell line (clone 2a) was maintained on hE-cad-Fc coated plates in mTeSR1 for a further 20 passages and found to maintain expression of pluripotency markers Oct4, SSEA4, and alkaline phosphatase activity (not shown). Following the 20 passages, the cells were introduced into Rag2^-/-^Il2rg^-/- ^mice and ten weeks after transplantation, teratomas containing tissues representative of all three germ layers were generated (Figure 5B). Based on these data we conclude that both huES cells and human iPS cells can be maintained in completely defined culture conditions using recombinant hE-cad-Fc as a substratum.

## Discussion

Although a number of defined media have been described that support pluripotent stem cell growth, most rely on the use of culture dishes coated with substrates that are relatively impure preparations of extracellular matrices. This report describes the use of highly pure recombinant hE-cad-Fc as a substitute for basement membrane that facilitates maintenance of pluripotency under completely defined conditions. Pluripotent stem cells cultured on an hE-cad-Fc-coated surface were virtually indistinguishable from those grown on a Matrigel-coated surface, including cell morphology, rate of proliferation, maintenance of an undifferentiated phenotype, and ability to differentiate into multiple cell types in both embryoid bodies and teratomas. The one difference was noted when cells were passaged using protease digestion cocktails such as Accutase, in that their adhesion efficiency was reduced; however, reduced adherence was circumvented by using enzyme-free Cell Dissociation Buffer to passage the cells. Indeed we believe that the use of enzyme-free methods to passage pluripotent stem cells has advantages, including the preservation of cell-surface proteins that can be used for FACS analyses and sorting.

Historically, integrin-mediated cell-ECM interactions have been considered essential for maintenance of stem cell pluripotency and viability [24-26]. Based on such studies, a significant effort has been devoted to finding a suitable ECM component that can maintain interactions with these cell surface receptors. In mouse ES cells, type I and IV collagen have been reported as contributing to the maintenance of pluripotency [27], although mouse ES cells don't appear to express α1ß1 or α2ß1 integrins, which are the predominant receptors through which cells adhere to collagen within a basement membrane. In addition, laminin-511 was also found to enable the maintenance of pluripotency of mouse ES cells through interactions with α6ß1 and α5ß1 integrins [28]. Recently recombinant vitronectin [29], collagen [30] and laminin-511 [31] have been reported as matrices that support human ES cells pluripotency all of which facilitate binding through interaction with integrins.

The absence of integrin binding proteins within the hE-cad-Fc substratum may indicate that integrin-mediated signalling is dispensable for stem cell maintenance. Integrin-ECM interactions predominantly act through integrin-linked kinase (ILK) or focal adhesion kinase (FAK) signalling pathways, and a subset of integrins have been shown to activate PI3K/Akt and MAPK pathways [32]. Although E-cadherin-mediated adhesion is commonly associated with ß-catenin signalling, it also stimulates PI3K/Akt signalling at least in ovarian carcinoma cells [33]. The Akt signalling pathway has been considered important for maintenance of pluripotency in mouse, primate, and human ES cells [34,35]. Although further analyses are required before the exact mechanism through which the hE-cad-Fc surface can support pluripotency is fully understood, we speculate that trans-homodimerization between cell surface E-cadherin and the E-cadherin domain presented through the hE-cad-Fc surface could facilitate stem cell maintenance by activation of PI3K/Akt signalling pathway. Consistent with this reasoning, several studies have implicated a role for E-cadherin in controlling both pluripotency and differentiation of ES cells [15,18,36], although the mechanism through which E-cadherin regulates these processes appears to be complex.

We previously reported that using mouse E-cad-Fc as a substratum, mouse ES cells, although retaining pluripotency, transition to a mesenchymal morphology and scatter across the plate rather than forming colonies [19,20]. In contrast, human pluripotent stem cells retain their ability to form colonies and show no evidence of undergoing an epithelial to mesenchymal transition (Figures 1B and 2A). The mechanisms underlying the different response to culture on E-cadherin substrate between mouse and human pluripotent stem cells have yet to be defined. However, human and mouse embryonic stem cells have been shown to display substantial differences in expression of transcription factors, cytokines, cell surface markers, growth factor receptors, and proteins involved in cell cycle control [37]. These differences in gene expression between mouse and human ES cells can often explain functional distinctions between the cell types, including the independence of human ES cells on LIF for maintaining pluripotency, the ability of human but not mouse ES cells to form trophectoderm in culture, and the relatively slow rate of proliferation of human ES cells compared to that of mouse. Harb *et al*. demonstrated that the inhibition of Rho-ROCK pathway induces cell scattering of human ES cells [38], indicating that there are different mechanisms that control cytoskeletal reorganization in human and mouse ES cells. Although both mouse and human ES cells express E-cadherin, it seems possible that a subset of the myriad of proteins that interact to define E-cadherin biological activity could differ between mouse and human ES cells, and experiments to address this are currently underway.

## Conclusions

In conclusion, we have demonstrated that hE-cad-Fc can support the viability and stem cell character of huES cells using completely defined culture conditions. We also found that like huES cells, hiPS cells could also be maintained on an hE-cad-Fc-coated surface. Progress toward generating hiPS cells that could be compatible with therapeutic procedures has been rapid. For example, several defined media that support reprogramming and hiPS culture without MEFs have been described, the avoidance of using selectable markers during reprogramming has been successful, and the need to use potentially mutagenic lentiviruses to convert somatic cells into hiPS cells has been circumvented. However, the availability of a defined substratum that avoids the possibility of animal or viral contaminants yet supports the generation and maintenance of hiPS cells has remained a challenge. We propose that recombinant hE-cad-Fc, with its ease of purification, simplicity of use, and its reproducibility, should allow the widespread replacement of Matrigel as a substratum for routine culture of human pluripotent stem cells.

## Methods

### Cell Culture

The generation of human iPS cells used in the current manuscript have been described in detail elsewhere [23] following the procedures established by Yu *et al*[3]. huES (H9 cells and H1 cells) and human iPS cells (hiPSC2a, hiPSC3a and hiPSC6a) were maintained on MEFs in DMEM/F12 (Invitrogen, Carlsbad, CA), supplemented with 1 mM L-glutamine (Millipore, Billerica, MA, http://www.millipore.com), 1% nonessential amino acids (Millipore, Billerica, MA), 0.1 μM ß-mercaptoethanol (Sigma Chemical, St. Louis, MO), 20% (vol/vol) Knockout serum replacement (KSR; Invitrogen, Carlsbad, CA) and 4 ng/mL bFGF. For feeder-free cultures, cells were cultured on a Matrigel-coated dish in medium conditioned by mitomycin C-treated MEF supplemented with 4 ng/mL bFGF or in mTeSR1 (Stemcell Technologies, Vancouver, Canada, http://www.stemcell.com). Cells were passaged with Accutase (Millipore, Billerica, MA) or enzyme-free Cell Dissociation Buffer (PBS-based: Invitrogen, Carlsbad, CA) before becoming confluent by incubating at room temperature until cells detached from the surface. For passage from Matrigel to hE-cad-Fc coated plates it was crucial to remove cells either using Cell Dissociation Buffer or minimal digestion with Accutase (<3 mins). All media contained 100 units/mL penicillin and 100 μg/mL streptomycin (Millipore, Billerica, MA). Cell karyotype and FISH analyses were performed by Cell Line Genetics, LLC (Madison, WI)

### Expression of fusion protein and preparation of hE-cad-Fc-coated dishes

To construct hE-cad-Fc, the cDNA that encodes human E-cadherin extracellular domain was amplified by nested PCR with KOD plus polymerase (TOYOBO, Osaka, Japan, http://www.toyobo.co.jp) from the cDNA of A431 cells. The specific primer pairs were used for amplification: 5'- AAG CAC CTG TGA GCT TGC G -3' and 5'- AAG TCC TGG TCC TCT TCT CCG C -3' for 1st PCR; 5'- AAG CTT CCA CCA TGG GCC CTT GGA GCC GCA GC -3' and 5'- GCG GCC GCT CTT CCT ACA GAC GCC GGC GGC CCC -3' for nested PCR. The cDNA of human E-cadherin fragment and mutated mouse IgG1 Fc domain (T252M-T254S) [39], which has a high affinity to Protein A, were ligated with pRC/CMV (Invitrogen, Carlsbad, CA) fragment, which was digested with *HindIII *and *XbaI *to generate the expression vector "pRC-hECFC." 293T cells were transfected with "pRC-hECFC" using Lipofectamine and PLUS reagent (Invitrogen, Carlsbad, CA) according to the manufacturer's directions. After selection of a highly expressing clone, 1C8, with 400 μg/mL G418 (Invitrogen, Carlsbad, CA), conditioned media were collected. The fusion proteins were loaded onto a rProtein A FF column (GE Healthcare Life Sciences, Pittsburgh, PA). The column was washed with 20 mM phosphate buffer (pH 7.0), and the bound proteins were eluted using 0.1 M sodium citrate (pH 2.7) followed by neutralization with a 1/5 volume of 1.0 M Tris-HCl (pH 9.0). Eluates were dialyzed against PBS containing 0.9 mM CaCl_2 _and 0.9 mM MgCl_2 _for 3 days. To prepare the hE-cad-Fc-coated surface, purified hE-cad-Fc solution was directly added to non-treated polystyrene plates at a concentration of 20 μg/ml unless otherwise described. After 2 h incubation at 37°C, plates were washed with PBS once and then cells were seeded.

### Alkaline phosphatase detection and immunocytochemistry

Alkaline phosphatase activity was determined using an alkaline phosphatase detection kit (Millipore, Billerica, MA). For immunocytochemistry, cells were fixed with 8% formaldehyde/PBS solution for 10 min and made permeable with 0.2% Triton X-100 (Fisher Scientific International, Hampton, NH, http://www.fishersci.com) for 2 min at room temperature. Fixed cells were incubated with 1% BSA/PBS for 1 h at room temperature and then stained with anti-mouse Oct-3/4 polyclonal antibody (H-134; Santa Cruz Biotechnology, Santa Cruz, CA, http://www.scbt.com), anti-phospho-histone H3 (Ser10) antibody (Millipore, Billerica, MA), or anti-activated caspase-3 antibody (BD Pharmingen, San Diego, CA, http://www.bdbiosciences.com) for 2 h, followed by Alexa Fluor-conjugated secondary antibodies (Invitrogen, Carlsbad, CA) for 1 h. Nuclei were counterstained with 0.5 μg/mL DAPI (Invitrogen, Carlsbad, CA). Samples were observed by fluorescence microscopy. For BrdU staining, cells were incubated with 10 μM BrdU (Sigma-Aldrich, St Louis) for 75 min at 37°C and then fixed and permeabilized using the same method as above. The nuclear DNA was denatured by incubating in 2N HCl for 30 min at room temperature. After washing with PBS, cells were stained with anti-BrdU antibody (clone BU1/75 ICR1; Abcam, Cambridge, U.K., http://www.abcam.com), followed by Alexa Fluor-conjugated secondary antibody, as above.

### Flow cytometry analysis

For analysis cell-surface SSEA-4 expression, cells were treated with Accutase (Millipore, Billerica, MA) and stained with phycoerythrin-conjugated anti-SSEA-4 antibody (Millipore, Billerica, MA). For E-cadherin expression, cells were treated with Accutase or Cell Dissociation Buffer and stained with phycoerythrin-conjugated anti-E-cadherin antibody (R&D systems, Minneapolis, MN, http://www.rndsystems.com). Stained cells were analyzed using Guava EasyCyte system (Millipore, Billerica, MA).

### RT-PCR analysis

Total RNA was isolated with RNeasy Plus Mini Kit (Qiagen, Valencia, CA, http://www.qiagen.com). First-strand cDNA was synthesized using Moloney murine leukemia virus (M-MLV) reverse transcriptase (Invitrogen, Carlsbad, CA), and PCR was carried out with Taq polymerase in supplier's reaction buffer containing 1.5 mM MgCl_2_. Oligonucleotide primer sequences are available by request. Amplicons were analyzed by 2% agarose gel electrophoresis.

### Adhesion and growth assays

For the adhesion assay, cells were seeded at a density of 1.0 × 10^5 ^cells/well into 24-well plates pre-coated with Matrigel, mouse IgG (Jackson Immunoresearch Laboratories, West Grove, PA, http://www.jacksonimmuno.com) or hE-cad-Fc. After 3 h of culture, medium and non-adherent cells were removed and cells were washed with culture medium. Adherent cells were stained with Cell Proliferation Reagent WST-1 (Roche Applied Science, Indianapolis, IN, http://www.roche-applied-science.com). After incubation for 2.5 h, absorbance at 450 nm was measured using a microplate reader (BioTek Instruments, Winooski, VT, http://www.biotek.com). For the cell growth assay, cells were seeded into a 96-well plate coated with hE-cad-Fc at a density of 3,000 cells/well or coated with Matrigel at a density of 1,000 cells/well. The cell number was evaluated once per day by the same method as for adhesion assay.

### Teratoma formation assay

Cells were collected by Accutase treatment and injected into immunocompromised Rag2^-/-^Il2rg^-/- ^mice (Taconic, Hudson, NY, http://www.taconic.com) subcutaneously or in the hind limb muscle. After 9 to 10 weeks, teratomas were surgically dissected from the mice. Samples were fixed in 4% zinc-formaldehyde and embedded in paraffin. Sections were cut at 4 μm and processed with hematoxylin and eosin staining. The Medical College of Wisconsin's IACUC approved all animal procedures used in this study.

### Western Blotting

Total protein was extracted with lysis buffer (10 mM Tris-HCl [pH 7.4], 150 mM NaCl, 1% Nonidet P40, 1% Triton X-100, 1 mM CaCl_2_). Samples were separated by electrophoresis on 12% polyacrylamide gels and electrophoretically transferred to a polyvinylidene difluoride membrane (Bio-Rad Laboratories, Hercules, CA, http://www.bio-rad.com). Blots were probed with anti-E-cadherin antibody (BD Transduction Laboratories, San Diego, CA, http://www.bdbiosciences.com) or anti-ß-actin antibody (Sigma), followed by horseradish peroxidase-conjugated secondary antibodies, and developed by SuperSignal West Pico substrate (Thermo Scientific, Waltham, MA, http://www.thermo.com).

### Coating efficiency of hE-cad-Fc onto polystyrene surface

Purified hE-cad-Fc protein was labelled with peroxidase labelling kit-NH_2 _(Dojindo Molecular Technologies, Inc., Rockville, MD). Labelled protein was diluted with unlabelled hE-cad-Fc (1/400) and then serially diluted with PBS. Diluted samples were added to a 96-well plate and incubated for 1 h at 37°C. Surfaces were washed with PBS three times and incubated with 100 μl of tetramethylbenzidine (TMB) substrate at room temperature. After addition of 100 μl of 1N HCl, absorbance at 450 nm was measured using a microplate reader. The amount of immobilized hE-cad-Fc was calculated from a standard curve of labelled hE-cad-Fc captured by anti-mouse IgG antibody-immobilized surface.

### Statistical analysis

Values are reported as means ± SD or SEM. Statistical significance was assessed using the paired Student's t-test. The probability level accepted for significance was P < 0.05.

## Authors' contributions

MN conducted the majority of experimental analyses and contributed to the writing of the manuscript. KST provided support and generated human iPS cells. TA provided plasmid, cells and advice for the purification hE-Cad-Fc fusion protein. SAD oversaw all aspects of the project and contributed to the writing of the manuscript. All authors read and approved the manuscript.

## References

[B1] Thomson JA Itskovitz-Eldor J Shapiro SS Waknitz MA Swiergiel JJ Marshall VS Jones JM Embryonic stem cell lines derived from human blastocysts Science 1998 282 1145 1147 10.1126/science.282.5391.1145 9804556

[B2] Takahashi K Tanabe K Ohnuki M Narita M Ichisaka T Tomoda K Yamanaka S Induction of pluripotent stem cells from adult human fibroblasts by defined factors Cell 2007 131 861 872 10.1016/j.cell.2007.11.019 18035408

[B3] Yu J Vodyanik MA Smuga-Otto K Antosiewicz-Bourget J Frane JL Tian S Nie J Jonsdottir GA Ruotti V Stewart R Slukvin II Thomson JA Induced pluripotent stem cell lines derived from human somatic cells Science 2007 318 1917 1920 10.1126/science.1151526 18029452

[B4] Lerou PH Daley GQ Therapeutic potential of embryonic stem cells Blood Rev 2005 19 321 331 10.1016/j.blre.2005.01.005 16275420

[B5] Stewart MH Bendall SC Bhatia M Deconstructing human embryonic stem cell cultures: niche regulation of self-renewal and pluripotency J Mol Med 2008 86 875 886 10.1007/s00109-008-0356-9 18521556

[B6] Xu C Inokuma MS Denham J Golds K Kundu P Gold JD Carpenter MK Feeder-free growth of undifferentiated human embryonic stem cells Nat Biotechnol 2001 19 971 974 10.1038/nbt1001-971 11581665

[B7] Kleinman HK McGarvey ML Liotta LA Robey PG Tryggvason K Martin GR Isolation and characterization of type IV procollagen, laminin, and heparan sulfate proteoglycan from the EHS sarcoma Biochemistry 1982 21 6188 6193 10.1021/bi00267a025 6217835

[B8] Kleinman HK McGarvey ML Hassell JR Star VL Cannon FB Laurie GW Martin GR Basement membrane complexes with biological activity Biochemistry 1986 25 312 318 10.1021/bi00350a005 2937447

[B9] Vukicevic S Kleinman HK Luyten FP Roberts AB Roche NS Reddi AH Identification of multiple active growth factors in basement membrane Matrigel suggests caution in interpretation of cellular activity related to extracellular matrix components Exp Cell Res 1992 202 1 8 10.1016/0014-4827(92)90397-Q 1511725

[B10] Kleinman HK Martin GR Matrigel: basement membrane matrix with biological activity Semin. Cancer Biol 2005 15 378 386 10.1016/j.semcancer.2005.05.004 15975825

[B11] Taub M Wang Y Szczesny TM Kleinman HK Epidermal growth factor or transforming growth factor alpha is required for kidney tubulogenesis in matrigel cultures in serum-free medium Proc Natl Acad Sci USA 1990 87 4002 4006 10.1073/pnas.87.10.4002 2339133 PMC54032

[B12] Carlson JA Garg R Compton SR Zeiss C Uchio E Poliomyelitis in SCID Mice Following Injection of Basement Membrane Matrix Contaminated with Lactate Dehydrogenase-elevating Virus Proceedings of the 59th AALAS National Meeting, PS35: 9-13 November 2008, Indianapolis

[B13] Takeichi M Morphogenetic roles of classic cadherins Curr Opin Cell Biol 1994 7 619 627 10.1016/0955-0674(95)80102-2 8573335

[B14] Gumbiner BM Regulation of cadherin-mediated adhesion in morphogenesis Nat Rev Mol Cell Biol 2005 6 622 634 10.1038/nrm1699 16025097

[B15] Larue L Antos C Butz S Huber O Delmas V Dominis M Kemler R A role for cadherins in tissue formation Development 1996 122 3185 3194 8898231 10.1242/dev.122.10.3185

[B16] Dang SM Gerecht-Nir S Chen J Itskovitz-Eldor J Zandstra PW Controlled, scalable embryonic stem cell differentiation culture Stem Cells 2004 22 275 282 10.1634/stemcells.22-3-275 15153605

[B17] Eastham AM Spencer H Soncin F Ritson S Merry CL Stern PL Ward CM Epithelial-mesenchymal transition events during human embryonic stem cell differentiation Cancer Res 2007 67 11254 11262 10.1158/0008-5472.CAN-07-2253 18056451

[B18] Ullmann U In't Veld P Gilles C Sermon K De Rycke M Van de Velde H Van Steirteghem A Liebaers I Epithelial-mesenchymal transition process in human embryonic stem cells cultured in feeder-free conditions Mol Hum Reprod 2007 13 21 32 10.1093/molehr/gal091 17090644

[B19] Nagaoka M Koshimizu U Yuasa S Hattori F Chen H Tanaka T Okabe M Fukuda K Akaike T E-cadherin-coated plates maintain pluripotent ES cells without colony formation PLoS ONE 2006 1 e15 10.1371/journal.pone.0000015 17183641 PMC1762325

[B20] Nagaoka M Hagiwara Y Takemura K Murakami Y Li J Duncan SA Akaike T Design of the artificial acellular feeder layer for the efficient propagation of mouse embryonic stem cells J Biol Chem 2008 283 26468 26476 10.1074/jbc.M805037200 18614540 PMC3258920

[B21] Ludwig TE Levenstein ME Jones JM Berggren WT Mitchen ER Frane JL Crandall LJ Daigh CA Conard KR Piekarczyk MS Llanas RA Thomson JA Derivation of human embryonic stem cells in defined conditions Nat Biotechnol 2006 24 185 187 10.1038/nbt1177 16388305

[B22] Rosler ES Fisk GJ Ares X Irving J Miura T Rao MS Carpenter MK Long-term culture of human embryonic stem cells in feeder-free conditions Dev Dyn 2004 229 259 274 10.1002/dvdy.10430 14745951

[B23] Si-Tayeb K Noto FK Nagaoka M Li J Battle MA Duris C North PE Dalton S Duncan SA Highly Efficient Generation of Human Hepatocyte-like Cells from Induced Pluripotent Stem Cells Hepatology 2009 51 297 305 10.1002/hep.23354 PMC2946078 19998274

[B24] Guilak F Cohen DM Estes BT Gimble JM Liedtke W Chen CS Control of stem cell fate by physical interactions with the extracellular matrix Cell Stem Cell 2009 5 17 26 10.1016/j.stem.2009.06.016 19570510 PMC2768283

[B25] Daley WP Peters SB Larsen M Extracellular matrix dynamics in development and regenerative medicine J Cell Sci 2008 121 255 264 10.1242/jcs.006064 18216330

[B26] Ilic D Culture of human embryonic stem cells and the extracellular matrix microenvironment Regen Med 2006 1 95 101 10.2217/17460751.1.1.95 17465823

[B27] Hayashi Y Furue MK Okamoto T Ohnuma K Myoishi Y Fukuhara Y Abe T Sato JD Hata R Asashima M Integrins regulate mouse embryonic stem cell self-renewal Stem Cells 2007 25 3005 3015 10.1634/stemcells.2007-0103 17717067

[B28] Domogatskaya A Rodin S Boutaud A Tryggvason K Laminin-511 but not -332, -111, or -411 enables mouse embryonic stem cell self-renewal in vitro Stem Cells 2008 26 2800 2809 10.1634/stemcells.2007-0389 18757303

[B29] Braam SR Zeinstra L Litjens S Ward-van Oostwaard D van den Brink S van Laake L Lebrin F Kats P Hochstenbach R Passier R Sonnenberg A Mummery CL Recombinant vitronectin is a functionally defined substrate that supports human embryonic stem cell self-renewal via alphavbeta5 integrin Stem Cells 2008 26 2257 2265 10.1634/stemcells.2008-0291 18599809

[B30] Furue MK Na J Jackson JP Okamoto T Jones M Baker D Hata R Moore HD Sato JD Andrews PW Heparin promotes the growth of human embryonic stem cells in a defined serum-free medium Proc Natl Acad Sci USA 2008 105 13409 13414 10.1073/pnas.0806136105 18725626 PMC2522264

[B31] Miyazaki T Futaki S Hasegawa K Kawasaki M Sanzen N Hayashi M Kawase E Sekiguchi K Nakatsuji N Suemori H Recombinant human laminin isoforms can support the undifferentiated growth of human embryonic stem cells Biochem Biophys Res Commun 2008 375 27 32 10.1016/j.bbrc.2008.07.111 18675790

[B32] Dedhar S Cell-substrate interactions and signaling through ILK Curr Opin Cell Biol 2000 12 250 256 10.1016/S0955-0674(99)00083-6 10712922

[B33] De Santis G Miotti S Mazzi M Canevari S Tomassetti A E-cadherin directly contributes to PI3K/AKT activation by engaging the PI3K-p85 regulatory subunit to adherens junctions of ovarian carcinoma cells Oncogene 2009 28 1206 1217 10.1038/onc.2008.470 19151754

[B34] Watanabe S Umehara H Murayama K Okabe M Kimura T Nakano T Activation of Akt signaling is sufficient to maintain pluripotency in mouse and primate embryonic stem cells Oncogene 2006 25 2697 2707 10.1038/sj.onc.1209307 16407845

[B35] Armstrong L Hughes O Yung S Hyslop L Stewart R Wappler I Peters H Walter T Stojkovic P Evans J Stojkovic M Lako M The role of PI3K/AKT, MAPK/ERK and NF-κB signalling in the maintenance of human embryonic stem cell pluripotency and viability highlighted by transcriptional profiling and functional analysis Hum Mol Genet 2006 15 1894 1913 10.1093/hmg/ddl112 16644866

[B36] Soncin F Mohamet L Eckardt D Ritson S Eastham AM Bobola N Russell A Davies S Kemler R Merry CL Ward CM Abrogation of E-cadherin-Mediated cell-cell contact in mouse embryonic stem cells results in reversible LIF-independent self-renewal Stem Cells 2009 27 2069 2080 10.1002/stem.134 19544408

[B37] Ginis I Luo Y Miura T Thies S Brandenberger R Gerecht-Nir S Amit M Hoke A Carpenter MK Itskovitz-Eldor J Rao MS Differences between human and mouse embryonic stem cells Dev Biol 2004 269 360 380 10.1016/j.ydbio.2003.12.034 15110706

[B38] Harb N Archer TK Sato N The Rho-Rock-Myosin signaling axis determines cell-cell integrity of self-renewing pluripotent stem cells PLoS One 2008 3 e3001 10.1371/journal.pone.0003001 18714354 PMC2500174

[B39] Nagaoka M Akaike T Single amino acid substitution in the mouse IgG1 Fc region induces drastic enhancement of the affinity to protein A Protein Eng 2003 16 243 245 10.1093/proeng/gzg037 12736366

